# MATLIGN: a motif clustering, comparison and matching tool

**DOI:** 10.1186/1471-2105-8-189

**Published:** 2007-06-08

**Authors:** Matti Kankainen, Ari Löytynoja

**Affiliations:** 1 Institute of Biotechnology, University of Helsinki, Helsinki, Finland; 2 EMBL-European Bioinformatics Institute, Hinxton, UK

## Abstract

**Background:**

Sequence motifs representing transcription factor binding sites (TFBS) are commonly encoded as position frequency matrices (PFM) or degenerate consensus sequences (CS). These formats are used to represent the characterised TFBS profiles stored in transcription factor databases, as well as to represent the potential motifs predicted using computational methods. To fill the gap between the known and predicted motifs, methods are needed for the post-processing of prediction results, i.e. for matching, comparison and clustering of pre-selected motifs. The computational identification of over-represented motifs in sets of DNA sequences is, in particular, a task where post-processing can dramatically simplify the analysis. Efficient post-processing, for example, reduces the redundancy of the motifs predicted and enables them to be annotated.

**Results:**

In order to facilitate the post-processing of motifs, in both PFM and CS formats, we have developed a tool called Matlign. The tool aligns and evaluates the similarity of motifs using a combination of scoring functions, and visualises the results using hierarchical clustering. By limiting the number of distinct gaps created (though, not their length), the alignment algorithm also correctly aligns motifs with an internal spacer. The method selects the best non-redundant motif set, with repetitive motifs merged together, by cutting the hierarchical tree using silhouette values. Our analyses show that Matlign can reliably discover the most similar analogue from a collection of characterised regulatory elements such that the method is also useful for the annotation of motif predictions by PFM library searches.

**Conclusion:**

Matlign is a user-friendly tool for post-processing large collections of DNA sequence motifs. Starting from a large number of potential regulatory motifs, Matlign provides a researcher with a non-redundant set of motifs, which can then be further associated to known regulatory elements. A web-server is available at .

## Background

Transcription factor mediated gene regulation is one of the main cellular mechanisms to control gene expression. The regulation is mostly performed by transcription factors binding onto short, degenerate sequence motifs that recur frequently in the genome [1,2]. The binding specificities of the factors are commonly summarised as position frequency matrices (PFM) or consensus sequences (CS): PFMs list the number of occurrences of each nucleotide (columns) across sites of aligned binding sites (rows), whereas CSs represent a motif sequence using a set of degenerate symbols that give each base decoded by the given symbol an equal frequency [2].

Computational methods are often used to predict gene regulatory elements from a set of promoter sequences of similarly behaving genes, e.g. a set of co-expressed genes. Since the regulatory elements targeted by a given transcription factor are expected to resemble each other, over-represented DNA elements are seen as an indication of a common regulatory element and searched for [3-5]. The actual motif discovery is performed using probabilistic or deterministic optimisation, or pattern enumeration techniques, which both – although for different reasons – report repetitive motifs. In the first case, the search algorithms may stochastically terminate at different solutions and, due to this ambiguity, repetition of the analysis is recommended and multiple sets of similar motifs are obtained [3]. A pattern enumeration technique that evaluates all possible patterns guarantees finding the most over-represented ones, but it also reports repetitive motifs as numerous overlapping forms of the same motif are discovered. On the other hand, regulatory elements are evolutionary restrained across species and gene regulation can, alternatively, be inferred by searching for conserved DNA segments [6]. In terms of motif discovery, some methods have recently been developed that incorporate evolutionary information in the search of enriched motifs; these methods, however, also report redundant sets of motifs as they typically use probabilistic or deterministic optimisation.

Motif prediction tools typically output sets of PFMs or CSs. Of these, CSs could be analysed using conventional sequence alignment methods, such as those in the Emboss-package [7], but these tools are not designed for analyses of hundreds of motifs and, hence, are inconvenient to use. Methods specifically designed to align and compare sequence motifs do exist, e.g. the pattern assembly and comparison tools in RSA-tools, YSRA, TREG, MatCompare, CompareAce and PROCSE [4,8-14]. However, each of them lacks some desired features, such as alignment of motifs with variable-length spacers, analyses of sets consisting of both PFMs and CSs, creation of new alignments or discovery of optimal and non-redundant motif sets.

**Figure 1:**
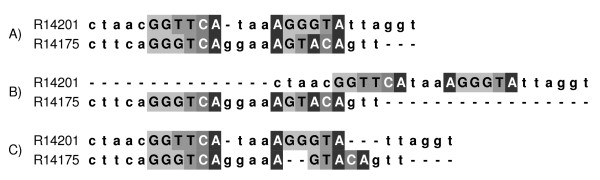
**Alignment of two cis-elements of the PRX:RXRa heterodimer**. Alignment of two cis-elements of the PRX:RXRa heterodimer using (A) Matlign with its default parameters, and Emboss programs needle/water (B) with no internal gaps and (C) with an unlimited number of gaps. Sites important for the DNA-protein interaction as given in TRANSFAC are capitalised (Acc. Number: R14201 and R14175) [17].

In order to combine all the necessary functions into a single framework, we developed a new tool called Matlign. Matlign accepts both CSs and PFMs as input, aligns these using a dynamic programming algorithm with a user-defined combination of scoring functions and an upper-bound for the number of internal gap events (0 to 1 in the web-server), and creates a graphical visualisation of the motif similarities using hierarchical clustering. If requested, the best non-redundant motif set is selected by cutting the tree according to the silhouette values and the false discovery rate (FDR) of matched motifs is controlled using a permutation-based method. The upper-bound for the number of distinct gaps ensures that motifs consisting of two half-sites separated by variable-length spacers, such as the regulatory elements of the medically important nuclear hormone receptors, are correctly aligned. For example, the alignment of the two divergent binding sites of the PRX:RXRa heterodimer [15-17] is erroneous if internal gaps are not allowed or if the number of gaps is unlimited (Figure 1). Naturally, an upper limit for the number of gaps does not prevent the algorithm from finding a better alignment without a gap if it exists.

In summary, Matlign is a practical post-processing tool for the comparison and clustering of short DNA motifs. We believe that Matlign is useful for tasks that involve measuring the similarity of motifs, such as identification of consensus motifs in multiple result sets, identification of the best matching hit from a collection of known regulative elements, and grouping together similar and redundant motifs. By reducing the undesired redundancy of raw data, Matlign saves the user from laborious and time-consuming analyses and facilitates the interpretation of motif prediction results.

## Implementation

We have implemented a dynamic programming algorithm for the alignment of motifs containing at most one internal gap event. Following the method of Gotoh [18], the match state is separated from the two gap states to allow for a more realistic gap cost function. However, similar to Sankoff [19], a return from a gap state to the earlier match state is not permitted and a move has to be taken to the succeeding match state. The procedure ensures that the chosen path has at most the specified number of distinct gap events but does not set a limit to the total length of gaps; terminal gaps are not penalised. See Additional file 1 for a more detailed description of the dynamic programming algorithm.

Matlign automatically converts CSs into PFMs and treats all input motifs in a similar manner. For CSs, the program supports the 15-letter IUPAC code and decodes the degenerate symbols to nucleotide frequencies by sharing the probability among relevant bases. This conversion can be adjusted by correcting the nucleotide frequencies according to a user defined AT/GC-ratio and/or by adding pseudocounts to matrices. Given the matrix representation of all motifs, the score of matching two motif sites is computed for two vectors of nucleotide frequencies. The scoring function for the motif matching can be chosen from the five implemented functions: Kendalls tau rank correlation coefficient (I), Spearman's rank correlation coefficient (II), Pearson correlation coefficient (III), normalised Euclidean distance (IV) and evolutionary substitution score (V), or any combination of these. Most of the functions are described in detail by Pietrokovski [13], who, for example, noted that the Spearman's and Pearson correlations are the most suitable functions for proteins [13]. Since CSs can be based on low frequency counts and their degenerate symbols produce somewhat artificial nucleotide frequencies, it is recommended to use the robust measures of correlation by the rank correlations for them. Matlign combines the different scores, or alternatively their Z-scores, by calculating their product and using a signum function that returns the most frequently occurring sign among the scores selected. When Z-scores are used, Matlign first estimates the population mean and standard deviation by performing an all-against-all matching of IUPAC symbols, i.e. each of the 15 symbols is matched against each other using the chosen distance function, and calculating the mean and standard deviation of these scores. The Z-scores are then derived by subtracting the population mean from an individual score and dividing the difference by the population standard deviation.

Agglomerative hierarchical clustering is a commonly-used method to group a collection of elements into subsets or clusters. It classifies the elements by recursively joining the two most similar ones, and creates a tree representing the nested grouping events (see the review of Jain et al., [20]). We start by computing an all-against-all similarity matrix of alignment scores using the dynamic programming algorithm. Then, the two most similar elements, i.e. the motif pair with the highest alignment score, are recursively joined until a single motif remains. In a joining event, a new motif is created as the alignment of the two motifs, and the distance matrix scores are updated by calculating the averaged distance between the motifs in the newly created cluster and all other motifs.

The optimal number of clusters from the hierarchically clustered tree is selected using silhouette-values [21]. The method describes the tightness and the separation of a clusters by calculating an average silhouette value *s*(*i*) of all the original elements:

s(i)=b(i)−a(i)max⁡[a(i),b(i)]
 MathType@MTEF@5@5@+=feaafiart1ev1aaatCvAUfKttLearuWrP9MDH5MBPbIqV92AaeXatLxBI9gBaebbnrfifHhDYfgasaacH8akY=wiFfYdH8Gipec8Eeeu0xXdbba9frFj0=OqFfea0dXdd9vqai=hGuQ8kuc9pgc9s8qqaq=dirpe0xb9q8qiLsFr0=vr0=vr0dc8meaabaqaciaacaGaaeqabaqabeGadaaakeaacqWGZbWCcqGGOaakcqWGPbqAcqGGPaqkcqGH9aqpdaWcaaqaaiabdkgaIjabcIcaOiabdMgaPjabcMcaPiabgkHiTiabdggaHjabcIcaOiabdMgaPjabcMcaPaqaaiGbc2gaTjabcggaHjabcIha4jabcUfaBjabdggaHjabcIcaOiabdMgaPjabcMcaPiabcYcaSiabdkgaIjabcIcaOiabdMgaPjabcMcaPiabc2faDbaaaaa@4C15@

The average distance from an element to all other elements within the same cluster *a*(*i*) is compared with the average distance from the element to the elements of the closest other cluster *b*(*i*). The resultant value is scattered between -1 (poor classification) and 1 (good classification) and the clustering yielding the highest average *s*(*i*) is chosen.

The false discovery rate (FDR) is the expected proportion of true null hypotheses rejected out of the total number of null hypotheses rejected [22]. In Matlign, the FDR for each alignment is calculated using a permutation technique. The alignment positions (rows) between the PFMs are first randomised, after which the nucleotide counts (columns) within each new randomised PFMs are separately permutated. Based on the desired number of these permutations, Matlign calculates the FDR for each alignment as the average number of permutated alignments that have a score as good or better than the real alignment's score, divided by the number of alignments in the real data that have a score as good or better than that score. As the process is repeated at each level of hierarchy, the calculation of FDR can be a time-consuming step.

## Results and discussion

Matlign is a tool to group and compare sequence motifs. To demonstrate the method's functionality, we describe a set of realistic examples of its usage. The first example focuses on the annotation of motifs, three following examples show how to use Matlign to reduce the redundancy of motif prediction results, and the last example how to create consensus predictions. The data for the examples presented here can be found and re-analysed using the Matlign server [23].

**Figure 2:**
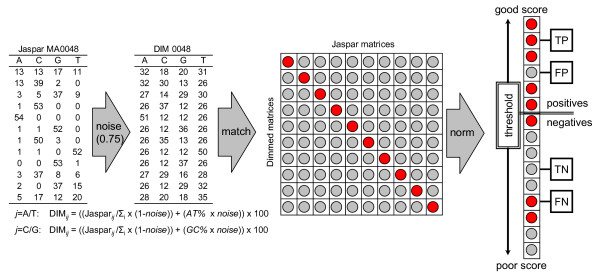
**Outline of the benchmarking test**. The diagram shows the benchmarking test used and an example of a noise-disturbed PFM with 75% noise added. In the pair-wise distance matrix in the middle, correct PFM pairs lay in the diagonal (red), all other are false pairs (grey). The panel on the right shows how true and false positives and negatives were assigned using a varying threshold. In the left panel, *i *is a row, *j *is a column in the matrix, *AT% *and *GC% *are the background AT/GC-content (34%/16%), and *noise *is the proportion of noise added.

Sequence motifs are often annotated by screening for similar motifs from a collection of known regulatory elements. To demonstrate how Matlign performs in this task, we downloaded all PFMs from the JASPAR database [24], replaced an increasing proportion of the nucleotide frequency signal with constant background noise, matched each noise-disturbed PFM against all the original PFMs, and performed a receiver operating characteristic (ROC) analysis (Figure 2). Before assessing the sensitivity and specificity, scores of each noise-disturbed PFM were scaled between 0 and 1. The sensitivity and specificity were calculated using varying score thresholds and all the predictions with a better score were considered as positives, either true or false, and all the predictions with a worse score as negatives, either true or false. The area under the ROC curve (AUC) was then determined for all the different distance functions and for their combinations (Additional file 2). Based on these results, the scoring scheme producing the best mean AUC over the data sets – the combination of Z-scores of Spearman's rank, Pearson correlation and evolutionary substitution score – was selected as the default scoring scheme.

**Figure 3:**
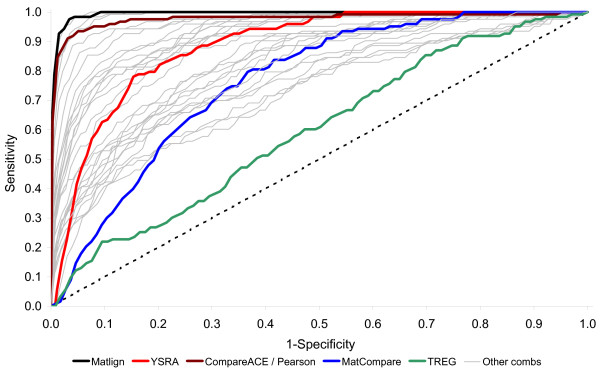
**Performance of different methods in the analysis of noise-disturbed data**. The performance of methods in the analysis of PFMs with 75% noise added. The ROC curves show the sensitivity (y-axis) and false positive rate, i.e. 1- specificity, (x-axis). Different combinations of the scoring functions of Matlign are shown in grey, with the best one highlighted in black. T-Reg (green) was run using default parameters and with the given orientation of the cis-elements [9]; MatCompare (blue) was run as recommended by the authors [11]. The performance of CompareACE/Pearson (brown) and YSRA (red) was simulated by implementing their corresponding distance functions in Matlign.

As the noise added to JASPAR PFMs increases (0–75%), they progressively resemble the average GC-content of the human genome and finally become almost indistinguishable from the background distribution. In real life, similar comparisons between perfect and noise-disturbed motifs occur when the predicted motifs are tainted by a partially incorrect input gene set or the poor performance of the prediction tool. The performance of Matlign compared to other methods tested suggests that Matlign outperforms its competitors and, at high noise levels, is more likely to find the correct PFM (Table 1 and Figure 3). Example 1 at the Matlign server shows the PFM data set with 75% of noise.

**Table 1:** Overall performance of different methods. Area under the ROC-curve (AUC) with different noise-disturbed data sets.

Program	Noise 0%	Noise 25%	Noise 50%	Noise 75%	Average
Matlign	1.00	1.00	1.00	0.99	1.00
CompareAce/Pearson	1.00	1.00	1.00	0.98	0.99
YSRA	1.00	1.00	1.00	0.88	0.97
MatCompare	1.00	1.00	0.97	0.76	0.93
TREG	0.99	0.99	0.90	0.60	0.87

Examples 2–4 demonstrate how to use Matlign for the post-processing of motifs obtained with different motif prediction tools. The test data, a set of promoter sequences of co-regulated genes from *S. cerevisiae*, was obtained from SCPD [25] by choosing the genes regulated by the PDR3 transcription factor. The first result set (Example 2) is from the probabilistic tool MotifSampler [3], whereas Examples 3 and 4 show the results of the pattern enumeration tools POCO and oligo-analysis [4,5]. In each example, Matlign is used to discover similar motifs from the redundant set produced by the corresponding motif prediction tool, to cluster these motifs together, and to return a non-redundant motif set to the user. In all analyses, Matlign was run using its default parameters (see Abbreviations for details).

In Example 2, at the highest silhouette value the original 100 predictions are grouped into 17 clusters that vary in size from a single motif to a cluster of 72 almost identical motifs. Using the same procedure, the 50 pattern predictions of POCO and oligo-analysis are reduced to 29 and 33 clusters, respectively. The number of clusters retained varies depending on the prediction method and indicates that certain tools can indeed remove a portion of the undesired redundancy. However, when the prediction results were post-processed using Matlign, these results were compressed to nearly one half of their original size, simplifying the analysis of the remaining motifs and the interpretation of the results.

**Figure 4:**
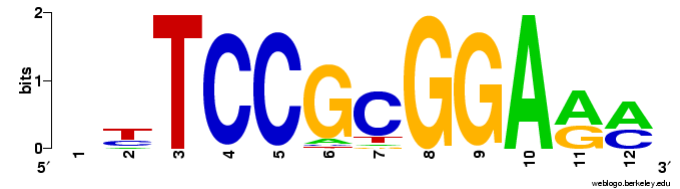
**Sequence logo of the consensus motif**. Sequence logo of the consensus motif created using WebLogo [27].

In Example 5, Matlign is used to perform a meta-analysis in order to see if the different tools agree on their predictions and if a representative consensus motif can be constructed out of the motifs they report. Similarly to the previous examples, the set of non-redundant motifs with the highest silhouette value was first selected and the motif with the highest over-representation was then chosen as the consensus motif. The motifs were evaluated using the program Clover [26] that calculates the over-representation of a PFM in a set of target promoters (here, the promoters of PDR3 regulated genes) relative to a collection of all yeast promoters; the method was chosen because it, unlike other similar tools, does not require an artificial PFM similarity threshold. The best motif (Figure 4) is found in the cluster that groups most individual motif predictions together and it matches well with the functional element of PDR3 given by SCPD [25]. It should be noted, however, that each motif prediction tool was able to predict motifs resembling the functional one and MotifSampler also found the true consensus pattern, although with a lower score and rank. However, that the original motifs from individual tools obtained less significant likelihood ratios in the Clover analysis demonstrates that post-processing with Matlign was able to improve the quality of motifs predicted (Table 2).

**Table 2:** Evaluation of best motifs. Best motifs predicted by Matlign and the individual motif prediction tools. *Rank *and *Hits *indicate the rank of the motif in the original analysis and the number of motif instances with raw score higher than or equal to 6, respectively. The significance of the motifs was determined using Clover [26].

Program	Rank	Consensus	Hits	Raw score	P-value
Matlign, node_nro119	-	AYTCCGCGGARM	19	40.8	<0.0001
MotifSampler	44	TCCGyGGA	24	31.9	<0.0001
oligo-analysis	2	CCGCGGAA	9	29.6	<0.0001
POCO	37	TCCGNGGA	24	27.1	<0.0001

## Conclusion

Matlign is a user-friendly web-tool to cluster and compare DNA sequence motifs. We have demonstrated that Matlign outperforms other available tools in finding remote analogues and is the preferable choice for the annotation and verification of potential binding site targets using collections of known motifs. By efficiently reducing the undesired redundancy of input motifs, Matlign speeds up the refinement of large collections of motif predictions and facilitates the interpretation of the results.

## Availability and requirements

Project name: Matlign

Project home page: 

Operating system: Unix

Programming language: C++/Perl

Other requirements: Following C++ libraries: studio, stdlib, string, vector, cmath, iostream, fstream, utility, cassert and config. For the web-server version: Zope, Gnuplot

License: GNU

Restrictions to use by non-academics: None

## Abbreviations

TFBS = transcription factor binding site

PFM = position frequency matrix

CS = consensus sequence

PXR = pregnane X receptor

RXRa = retinoic acid receptor alpha

FDR = false discovery rate

ROC = receiver operating characteristic

AUC = area under the receiver operating characteristic-curve

Default parameters: match = 5, transversion = -4, transition = -4, gap open = -10, gap extension = -1, maximal gap = undefined, spacers = true, Z-score = true, pseudocounts = 0, AT-frequency = 0.5, and Spearman's rank correlation coefficient, Pearson correlation coefficient and evolutionary substitution score (Viterbi-score)

## Authors' contributions

Both MK and AL were involved in developing and implementing the idea and writing this manuscript.

## Supplementary Material

Additional File 1 **Dynamic programming algorithm**. A detailed description of the dynamic programming algorithm Click here for file

Additional File 2 **The complete AUC data table**. Table showing the AUC values for all the different distance functions and for all noise-disturbed data sets. Click here for file
