# Supplementary material for: MATLIGN: a motif clustering, comparison and matching tool
Source: BMC Bioinformatics. 2007 Jun 8;8:189. doi: 10.1186/1471-2105-8-189 (PMC1925120; doi:10.1186/1471-2105-8-189)
Supplement: Additional File 1 — Dynamic programming algorithm. A detailed description of the dynamic programming algorithm [file 1471-2105-8-189-S1.doc]

**Dynamic programming algorithm**

We have implemented dynamic programming algorithm for the alignment of motifs containing at most one internal gap event. Let *s1* and *s2* be two sequences or matrices with lengths *n* and *m*, *k* be the maximum number of internal gap events, and *t* be the maximum length of terminal gaps. Given gap opening and extension parameters δ and ε, and scoring function s(i, j) to match two characters or character vectors (sites *i* and *j* in *s1* and *s2*, respectively), we can define the recursion to compute the optimal alignment:

Initialisation: *i*=0, ...,*n*, *j*=0, ...,*m* and *h*=1, ..., *k*;

$$M_{h}\left(i,j\right)=X_{h}\left(i,j\right)=Y_{h}(i,j)=M_{h+1}(i,j)=-\infty$$

then: *u*=0, ..., *t*;

$$M_{1}\left(\text{0,}u\right)=M_{1}\left(u,0\right)=0$$

Recursion: *i*=0, ...,*n*, *j*=0, ...,*m* except (0, 0), and *h*=1, ..., *k*;

$$\begin{matrix} M_{1}\left(i,j\right)=M_{1}\left(i-\text{1,}j-1\right)+s(i,j); \\ X_{h}\left(i,j\right)=\text{max}\left[M_{h}\left(i-\text{1,}j\right)+\delta,X_{h}\left(i-\text{1,}j\right)+\varepsilon\right]; \\ Y_{h}\left(i,j\right)=\text{max}\left[M_{h}\left(i,j-1\right)+\delta,Y_{h}\left(i,j-1\right)+\varepsilon\right]; \\ M_{h+1}\left(i,j\right)=\text{max}\left[X_{h}\left(i-\text{1,}j-1\right),Y_{h}\left(i-\text{1,}j-1\right),M_{h+1}\left(i-\text{1,}j-1\right)\right]+s(i,j); \end{matrix}$$

Termination: *u*=0, ..., *t*, and *h*=1, ..., *k*+1† or *h*=*k*+1‡;

$$M=\text{max}\left[M_{h}\left(n,m-u\right),M_{h}\left(n-u,m\right)\right].$$

(an upper-bound† and a fixed‡ number of gap events)
