# Supplementary material for: MATLIGN: a motif clustering, comparison and matching tool
Source: BMC Bioinformatics. 2007 Jun 8;8:189. doi: 10.1186/1471-2105-8-189 (PMC1925120; doi:10.1186/1471-2105-8-189)
Supplement: Additional File 2 — The complete AUC data table. Table showing the AUC values for all the different distance functions and for all noise-disturbed data sets. [file 1471-2105-8-189-S2.doc]

**Table 1.** Area under the ROC-curve in different noise disturbed data sets. In the table, *z* means that the z-score forms of the distance functions were used and *m* means the non-standardised values were multiplied. Kendall tau rank (I), Spearman’s rank (II), Pearson correlation (III), normalized Euclidean distance (IV) and evolutionary substitution score (V).

| rank | Method | noise 0% | noise 25% | noise 50% | noise 75% | average |
| --- | --- | --- | --- | --- | --- | --- |
| **1** | **Matlign z(II,III,V)** | **1.00** | **1.00** | **1.00** | **0.99** | **1.00** |
| 2 | z(V) | 1.00 | 1.00 | 1.00 | 0.99 | 1.00 |
| 3 | z(II,V) | 1.00 | 1.00 | 1.00 | 0.99 | 1.00 |
| 4 | z(III,V) | 1.00 | 1.00 | 1.00 | 0.99 | 1.00 |
| **5** | **CompareAce / Pearson** | **1.00** | **1.00** | **1.00** | **0.98** | **0.99** |
| 6 | z(II,III) | 1.00 | 1.00 | 1.00 | 0.97 | 0.99 |
| 7 | m(II,III) | 1.00 | 1.00 | 1.00 | 0.97 | 0.99 |
| 8 | m(II) | 1.00 | 1.00 | 1.00 | 0.96 | 0.99 |
| 9 | z(II) | 1.00 | 1.00 | 1.00 | 0.96 | 0.99 |
| 10 | m(I,III) | 1.00 | 1.00 | 1.00 | 0.96 | 0.99 |
| 11 | z(I,V) | 1.00 | 1.00 | 0.99 | 0.96 | 0.99 |
| 12 | m(I) | 1.00 | 1.00 | 0.99 | 0.95 | 0.98 |
| 13 | z(I,III,V) | 1.00 | 1.00 | 0.99 | 0.95 | 0.98 |
| 14 | m(I,II,III) | 1.00 | 1.00 | 1.00 | 0.93 | 0.98 |
| 15 | m(I,II) | 1.00 | 1.00 | 0.99 | 0.94 | 0.98 |
| 16 | z(IV,V) | 1.00 | 1.00 | 1.00 | 0.93 | 0.98 |
| 17 | z(III,IV,V) | 1.00 | 1.00 | 1.00 | 0.92 | 0.98 |
| 18 | z(I,II,V) | 1.00 | 0.99 | 0.98 | 0.94 | 0.98 |
| 19 | z(I,III) | 1.00 | 1.00 | 1.00 | 0.91 | 0.98 |
| 20 | m(II,III,V) | 1.00 | 1.00 | 1.00 | 0.91 | 0.98 |
| 21 | z(II,IV,V) | 1.00 | 1.00 | 1.00 | 0.91 | 0.98 |
| 22 | z(I,II,III,V) | 1.00 | 0.99 | 0.98 | 0.92 | 0.98 |
| 23 | z(II,III,IV,V) | 1.00 | 1.00 | 1.00 | 0.89 | 0.97 |
| 24 | z(I,II) | 1.00 | 1.00 | 0.99 | 0.90 | 0.97 |
| 25 | z(I,II,III) | 1.00 | 1.00 | 0.99 | 0.89 | 0.97 |
| 26 | m(I,III,V) | 1.00 | 1.00 | 1.00 | 0.88 | 0.97 |
| **27** | **YSRA** | **1.00** | **1.00** | **1.00** | **0.88** | **0.97** |
| 28 | m(I,II,III,V) | 1.00 | 1.00 | 1.00 | 0.85 | 0.96 |
| 29 | z(III,IV) | 1.00 | 1.00 | 1.00 | 0.84 | 0.96 |
| 30 | m(I,II,V) | 1.00 | 1.00 | 0.99 | 0.84 | 0.96 |
| 31 | m(III,IV) | 1.00 | 1.00 | 1.00 | 0.83 | 0.96 |
| 32 | z(I,IV,V) | 1.00 | 1.00 | 0.98 | 0.85 | 0.96 |
| 33 | z(IV) | 1.00 | 1.00 | 0.99 | 0.82 | 0.95 |
| 34 | z(II,IV) | 1.00 | 1.00 | 1.00 | 0.81 | 0.95 |
| 35 | m(IV) | 1.00 | 1.00 | 0.99 | 0.81 | 0.95 |
| 36 | z(I,III,IV,V) | 1.00 | 1.00 | 0.98 | 0.83 | 0.95 |
| 37 | m(II,IV) | 1.00 | 1.00 | 1.00 | 0.80 | 0.95 |
| 38 | z(I,II,IV,V) | 1.00 | 0.99 | 0.97 | 0.82 | 0.95 |
| 39 | z(II,III,IV) | 1.00 | 1.00 | 1.00 | 0.78 | 0.94 |
| 40 | m(II,III,IV) | 1.00 | 1.00 | 1.00 | 0.77 | 0.94 |
| 41 | z(I,II,III,IV,V) | 1.00 | 0.99 | 0.96 | 0.80 | 0.94 |
| 42 | m(I,IV) | 1.00 | 1.00 | 0.99 | 0.77 | 0.94 |
| 43 | z(I) | 1.00 | 1.00 | 0.98 | 0.78 | 0.94 |
| 44 | z(I,IV) | 1.00 | 1.00 | 0.98 | 0.76 | 0.94 |
| **45** | **MatCompare** | **1.00** | **1.00** | **0.97** | **0.76** | **0.93** |
| 46 | m(I,III,IV) | 1.00 | 1.00 | 0.99 | 0.74 | 0.93 |
| 47 | m(III,IV,V) | 1.00 | 1.00 | 1.00 | 0.73 | 0.93 |
| 48 | m(II,IV,V) | 1.00 | 1.00 | 1.00 | 0.71 | 0.93 |
| 49 | m(I,II,IV) | 1.00 | 1.00 | 0.98 | 0.73 | 0.93 |
| 50 | m(I,II,III,IV) | 1.00 | 1.00 | 0.98 | 0.72 | 0.93 |
| 51 | z(I,III,IV) | 1.00 | 1.00 | 0.98 | 0.73 | 0.93 |
| 52 | m(II,III,IV,V) | 1.00 | 1.00 | 1.00 | 0.71 | 0.93 |
| 53 | z(I,II,IV) | 1.00 | 1.00 | 0.97 | 0.73 | 0.92 |
| 54 | z(I,II,III,IV) | 1.00 | 1.00 | 0.97 | 0.72 | 0.92 |
| 55 | m(I,IV,V) | 1.00 | 1.00 | 1.00 | 0.68 | 0.92 |
| 56 | m(I,III,IV,V) | 1.00 | 1.00 | 1.00 | 0.67 | 0.92 |
| 57 | m(I,II,IV,V) | 1.00 | 1.00 | 0.99 | 0.66 | 0.91 |
| 58 | m(I,II,III,IV,V) | 1.00 | 1.00 | 0.99 | 0.65 | 0.91 |
| **59** | **TREG** | **0.99** | **0.99** | **0.90** | **0.60** | **0.87** |
| 60 | m(III,V) | 0.99 | 0.97 | 0.89 | 0.58 | 0.86 |
| 61 | m(II,V) | 0.99 | 0.97 | 0.89 | 0.54 | 0.85 |
| 62 | m(I,V) | 0.99 | 0.95 | 0.87 | 0.54 | 0.84 |
| 63 | m(V) | 0.99 | 0.97 | 0.82 | 0.45 | 0.81 |
| 64 | m(IV,V) | 1.00 | 0.96 | 0.75 | 0.48 | 0.80 |
